# Associations of peer generational status on adolescent weight across Hispanic immigrant generations: A social network analysis

**DOI:** 10.1016/j.socscimed.2023.115831

**Published:** 2023-03-13

**Authors:** Christopher J. Gonzalez, Molly Copeland, Martin F. Shapiro, James Moody

**Affiliations:** a Department of Medicine, Weill Medical College of Cornell University, 525 E 68th St., New York, NY, 10065, USA; b Department of Sociology, Michigan State University, East Lansing, MI, 48824, USA; c Department of Sociology, Duke University, Durham, NC, 27708, USA

**Keywords:** Weight, Social network, Hispanic, Acculturation

## Abstract

**Background:**

Childhood obesity disproportionately impacts Hispanics in the United States (US), the nation’s largest ethnic minority population. However, even among Hispanic children, those born in the US are at increased risk of developing obesity than those not born in the US (i.e. first-generation Hispanics). The objective of this study is to assess whether ethnic and generational differences in the friend networks of Hispanic adolescents moderate the association between immigrant generation and weight.

**Methods:**

We analyzed data from first-generation, second-generation, and third-generation Hispanic 12 to 19 year-old participants in Wave 1 of the Longitudinal Study of Adolescent to Adult Health (Add Health). Using multivariable linear regression, we examined the association between generational status and body mass index (BMI), and whether the ethnic and generational composition of friends moderated that association.

**Results:**

Higher generational status was associated with higher BMI. The ethnic and generational composition of friends was not independently associated with BMI among Hispanic adolescents. However, a social network with a greater proportion of second-generation Hispanics was positively associated with BMI among first-generation Hispanics, and negatively associated with BMI among second-generation Hispanics.

**Conclusions:**

The generational status of peers in Hispanic adolescents’ social networks, particularly the proportion that are second-generation Hispanic, moderates the positive association between immigrant generation and BMI. Moreover, this moderation effect is different across immigrant generations so that the proportion of second-generation adolescents within a social network is associated with higher BMI in first-generation Hispanic adolescents, but with lower BMI among those who are second-generation. These results were confirmed in sensitivity analyses. Our findings suggest that the generational composition of social networks alters the association between the generational status and weight of Hispanic adolescents, and thus that social factors within those networks may contribute to those associations.

## 1. Background

The health of Hispanics, the largest ethnic minority population in the United States (US), is growing disproportionately poor relative to that of non-Hispanic whites, particularly as it relates to obesity and obesity-related conditions like atherosclerotic disease and diabetes (Center, 2015; Kirk et al., 2008; Ogden et al., 2020; Velasco-Mondragon et al., 2016). These inequities become apparent at an early age: the prevalence of obesity among Hispanic children 2–19 years old is nearly twice that of their non-Hispanic white counterparts (Hales et al., 2017). Considering the long-term cardiovascular impact of childhood obesity, as well as its numerous psychosocial consequences including discrimination and weight-based victimization, the disproportionate rate of obesity among Hispanic children is a public health issue that requires attention (Beck, 2016; Burke et al., 2008; K. Liu et al., 2012b; Morrison et al., 2015; Serdula et al., 1993).

Notably, weight-related outcomes vary substantially even within the Hispanic population. First-generation Hispanic Americans (those born outside of the US), exhibit better cardiovascular risk profiles than subsequent generations of Hispanics (those that are US-born), including lower rates of obesity, more nutritional diets and lower rates of sedentary behaviors (M. L. Allen et al., 2007; Gordon-Larsen et al., 2003; McCullough and Marks, 2014; Popkin and Udry, 1998; Vishnu et al., 2019). These generational trends are often attributed to acculturation, which suggests that prolonged time in the US leads to the adoption of numerous mainstream behaviors, including the intake of highly-processed calorie-rich foods and the uptake of sedentary life-styles (Abraído-Lanza et al., 2016; Arcia et al., 2001; Karen R Flórez and Abraído-Lanza, 2017; Fox et al., 2017a; McLeod et al., 2016; Thomson and Hoffman-Goetz, 2009). Notably, acculturation a complex multi-dimensional concept and has been operationalized in a variety of ways, including nativity and generational status, which, while not direct measures of acculturation, consistently have been associated with weight (Karen R Flórez and Abraído-Lanza, 2017; Gordon-Larsen et al., 2003; Harris et al., 2009; McLeod et al., 2016; Popkin and Udry, 1998). Most importantly, the process by which immigrants adopt acculturated behaviors remains unclear, but prior research suggests that acculturation may be influenced by the social contexts that a person inhabits (Fox et al., 2017b). As an example, recent immigrants living in ethnic enclaves, geographic areas with a high concentration of individuals of a certain ethnicity, show less acculturated and more favorable weight-related outcomes than those living outside of those enclaves (Do and Frank, 2020; Kershaw et al., 2013; Moloney and South, 2015; Park et al., 2011). Socio-cultural factors more proximal to an individual, such as the ethnic concentration of their personal friend groups (i.e. their social network), may influence the adoption of acculturated behaviors, but there is a lack of research to date that has explored these associations (J. D. Allen et al., 2014).

Social network theory posits that social relationships are relevant to transmitting information as well as enabling attitudinal and behavioral change (W. Liu et al., 2017; Valente et al., 2015). Prior social network analyses have shown that social networks are associated with health behaviors and outcomes, including those related to weight like physical activity and obesity (Christakis and Fowler, 2007; Umberson and Montez, 2010). Social network peers are particularly important to shaping health behaviors among adolescents (Christakis and Fowler, 2007, 2008). Teens’ social ties can foster engagement in physical or sedentary activities or other explicitly weight-controlling behaviors, and teens may befriend peers given shared interests that contribute to health and weight, so that social networks become associated with weight and weight-related behaviors (de la Haye et al., 2010; Mueller et al., 2010). Obesity is also a visible, highly stigmatized characteristic in adolescence that may affect teens’ decisions regarding which peers to befriend or which friends to maintain, so that obesity itself may shape social networks (Crosnoe et al., 2008; Mueller et al., 2010; Schaefer and Simpkins, 2014).

Given ethnic and generational patterns in weight and weight-related behaviors, Hispanic adolescents may face unique weight-related social pressures within their social networks. For example, given the inherently social dynamics of acculturation, the ethnicity or immigrant generation of Hispanic adolescents’ friends may impede or facilitate the adoption of certain weight behaviors that in turn impact weight. In this way, adolescent social networks may shape the acculturation process related to weight for Hispanic youth.

The objective of the current study was to assess whether generational weight trends in Hispanic youth are moderated by the ethnicity and generational status of friends within their social networks. Consistent with prior literature, we hypothesize that (1) the body mass index (BMI) of Hispanic adolescents will increase across immigrant generations (i.e., second and third-generation Hispanic adolescents will have higher BMI than those who are first-generation) and (2) the ethnicity and generational status of friends will moderate the relationship between an adolescent’s own generational status and weight, so that social networks with a higher proportion of Hispanics and a higher proportion of first-generation Hispanics will attenuate the association between generational status and BMI.

## 2. Methods

### 2.1. Survey design

The study population consists of 90,118 adolescents enrolled in the US Longitudinal Study of Adolescent to Adult Health (Add Health). Add Health is a nationally representative study of US adolescents in grades 7 through 12, supplemented with subsamples of specific ethnic minority groups, including Hispanics, and collected under protocols approved by the Institutional Review Board of the University of North Carolina. Wave 1 took place between 1994 and 1995 and consisted of a 45-min questionnaire administered in the school. Of those completing the In-School questionnaire, 20,745 adolescents completed an additional In-Home interview. Our core analytic sample consisted of 2264 adolescents who completed the In-Home survey and had complete ethnicity data indicating they identified as Hispanic. We specifically selected the In Home sample for our analyses because data regarding weight and height were only collected in the In Home survey, and not in the school questionnaires. The survey design and sampling frame for Add Health have been described elsewhere (Popkin and Udry, 1998).

### 2.2. Measures

#### 2.2.1. Independent variable: Adolescent ethnicity and generational status

Race and Hispanic ethnicity of respondents was based on self-classification when surveyed during the In-Home survey. Hispanic ethnicity was based on the response to the question “Are you of Hispanic origin?” Nativity was categorized into two groups, (1) foreign-born and (2) US-born, based on the question “Were you born in the US?” Those not born in the US were categorized as foreign-born, those born in the US were categorized as US-born. Generational status was categorized into three groups, (1) first-generation, (2) second-generation and (3) third-generation and beyond, based on the nativity of the respondent and the respondent’s parents, based on the question “Was your biological parent born in the United States?” Foreign-born respondents were identified as first-generation, US-born respondents with at least one foreign-born parent as second-generation, and US-born respondents with US-born parents as third-generation (Afable-Munsuz et al., 2013; M. L. Allen et al., 2007; Popkin and Udry, 1998; Reingle et al., 2011). Responses from the Wave 1 In-Home surveys were used when available and supplemented with responses from the In-School survey when unavailable.

#### 2.2.2. Dependent variable: Adolescent body mass index (BMI)

There are several measures of weight and obesity; BMI is correlated with adiposity and its use is recommended by The American Academy of Pediatrics to screen for potential weight and health-related issues because it is independently associated with cardiometabolic risk. BMI was calculated in kg/m^2^ using respondent-reported weight and height at the time of the Wave 1 In-Home survey.

#### 2.2.3. Moderator: social network ethnicity and generational status of the peer social network

Respondents of the Add Health In School Questionnaire were asked to nominate up to 10 of their closest friends (up to 5 male and 5 female). The total size of an adolescent’s social network was calculated using the number of friends nominated by the respondent as well as the number of adolescents that nominated the respondent as a friend. Ethnicity and generational status of respondents in the social network was based on self-reported Hispanic ethnicity, nativity, and parental nativity as above. To assess the associations of network characteristics relative to network size, the social network variables of interest were calculated as a proportion of each respondent’s total social network. Ethnic density of the social network was calculated as the proportion of the network that identified as Hispanic. Generational density was calculated as three separate variables: the proportion of the total social network that identified as first-, second- and third-generation Hispanic, respectively. The objective was to assess network composition, thus isolates, individuals with a social network size of 0, were not included in proportion analyses.

#### 2.2.4. Additional covariates

Consistent with prior literature assessing the role of generational status on weight and weight-related behaviors in adolescent populations, we adjusted our models for sex, age, and socioeconomic status, using parental receipt of public assistance as a proxy (M. L. Allen et al., 2007; Gordon-Larsen et al., 2002; Gordon-Larsen et al., 2003; J. Liu et al., 2012a; Richmond et al., 2007; Richmond et al., 2006).

Physical activity was measured as the self-reported number of times per week that adolescents performed 3 groups of physical activity. These three groups of activities were asked in 3 separate questions as “During the past week how many times did you …” and included 1) riding bike, roller-skating or rollerblading, 2) participation in active sports such as soccer, baseball, or basketball, and 3) active exercises such as walking, running, dancing or jumping rope. These measures were chosen as part of the survey because of their qualification as moderate to vigorous physical activity (MVPA; 5 to 8 metabolic equivalents), and the use of similar validated questions in other assessments of physical activity (Godin, 2011; Gordon-Larsen et al., 2003; Sallis et al., 1993). Because activities included within each individual question require a variety of metabolic equivalents, the precise metabolic equivalent value of a question could not be calculated. Possible responses were categorical (not at all, 1–2 times, 3–4 times, or >5 times), precluding calculations of hours per week performing physical activity. Consistent with prior literature quantifying physical activity in Add Health, we calculated a summation of times per week engaged in moderate to vigorous physical activity (Richmond et al., 2006). This method assigns a numerical value to each categorical response, using the mean of the range of that response (i.e., 1–2 times is assigned 1.5) (Richmond et al., 2006).

Sedentary activity was measured as the self-reported hours per week that adolescents performed 3 activities: 1) viewing television, 2) viewing video cassettes and 3) computer/video game use (Gordon-Larsen et al., 2002, 2003). These activities are typically performed seated, require less than 1.5 metabolic equivalents, and are typically utilized as measures of sedentary activity (M. L. Allen et al., 2007; Hidding et al., 2017). Consistent with prior work, a summation approach was used to estimate the total number of hours per week engaged in sedentary time (Gordon-Larsen et al., 2003).

### 2.3. Statistical analysis

We used linear regression to model the association of adolescents’ generational status with BMI. The initial model (Model 1) controlled solely for sociodemographic variables: age, sex, and parental receipt of public assistance. We then assessed the social network characteristics of interest by adding them in a step-wise approach to this initial model.

Model 2 and 3 assessed for associations between the ethnic density of the peer social network and BMI. Specifically, Model 2 included the proportion of the network that identified as Hispanic as an additional covariate. To assess whether the ethnic composition of a network is associated with weight differently across Hispanic generations, Model 3 assessed interactions between the proportion of the network that identified as Hispanic and the adolescent’s own generational status.

Models 4 through 7 assessed whether the generational density of the peer social network predicted BMI. Specifically, following a similar analytic method, Model 4 added three social network covariates to the initial model: the proportion of the network that was each first-, second- and third-generation Hispanic. Models 5 through 7 assessed for interactions between the adolescent’s generational status and the proportion of the network that identified as first-, second- and third-generation Hispanic, respectively. Finally, to assess whether any network related associations were mediated by weight-related behaviors, models with statistically significant interactions (p < 0.05) were then additionally controlled for physical and sedentary activity. All models that included social network variables were additionally controlled for the total size of the network to independently account for network size. All analyses were conducted in Stata 16. Additionally, robust standard errors were used in all analyses (Huber, 1967; Rencher and Christensen, 2012).

Sensitivity analyses using multiple imputation (with multiple imputation with chained equations in Stata) to fill missing covariate data among participants in our core analytic sample, provided in Supplement Table 1 (StataCorp, 2021). While prior studies have imputed BMI on the full Add Health sample, BMI was our primary outcome, precluding our ability to expand our core analytic sample (Schaefer and Simpkins, 2014).

## 3. Results

Among 2264 Hispanic adolescents in W1 of Add Health (1950) had complete data. 543 (27.9%) were first-generation, 916 (47.0%) were second-generation, and 491 (25.2%) were third-generation. The mean age of Hispanic adolescents was 16 years old (SD 1.65), and the majority were male (50.6%). Mean BMI was 23.20 kg/m^2^ (SD 4.54). Further descriptive details are available in Table 1.

In multivariable models (Table 2), when controlling for age, sex and socioeconomic status (***Model 1***), second and third-generation Hispanic adolescents were statistically more likely to have higher BMIs than those who were first-generation (β co-efficient 0.94, p < 0.001, and β co-efficient 0.88, p < 0.01, respectively). The ethnic density (i.e. proportion peers that were Hispanic) of the social network of Hispanic adolescents did not predict the adolescent’s BMI (***Model 2***), nor did it significantly interact with the generational status of the adolescent (***Model 3***).

The generational density (i.e. the proportion of peers that were first-, second- and third-generation Hispanic) of the social network of Hispanic adolescents also did not predict BMI overall (***Model 4***). However, there were statistically significant interactions between the proportion of second-generation Hispanic peers in the social network and an adolescent’s own generational status: a higher proportion of second-generation Hispanic peers in the social network was associated with a higher BMI among first-generation Hispanic adolescents (β co-efficient 1.64, p < 0.05) but with a lower BMI among second-generation Hispanic adolescents (β co-efficient −1.92, p < 0.05) (***Model 6***). There was no statistically significant interaction between an adolescent’s generational status and the proportions of first (***Model 5***) and third-generation (***Model 7***) Hispanics within their social networks. When adding behavioral covariates to the statistically significant model (***Model 8***), the interaction between adolescent’s generational status and the proportion of second-generation Hispanic peers within the social network remained significant. In this model, sedentary activity was positively associated with BMI, while MVPA was non-significant.

Results were unchanged in the sensitivity analysis of the imputed sample, with the exception that the interaction between adolescent’s generational status and the proportion of second-generation Hispanic peers within the social network became nonsignificant after adding behavioral covariates, specifically MVPA and sedentary activity (Supplementary Table 1).

## 4. Discussion

This study fills a key gap in understanding generational trends in weight among Hispanic adolescents by assessing whether those trends are moderated by friends’ ethnicity and generational status. We found that second and third-generation Hispanic adolescents had higher BMIs than those who were first-generation, even when controlling for sociodemographic variables. Moreover, these generational trends in BMI were not moderated by having Hispanic friends, but the generational status of Hispanic friends did matter, in ways that varied with the adolescent’s own generational status. Among first-generation Hispanics, a social network with relatively more second-generation Hispanic friends predicted a higher BMI, but among second-generation Hispanics, relatively more second-generation Hispanic friends predicted a lower BMI.

This study supports prior evidence that weight is higher in second and third-generation Hispanic adolescents than among those who are first-generation (Popkin and Udry, 1998) and that these generational trends remain significant even when adjusting for structural socioeconomic factors (Gordon-Larsen et al., 2003). However, the current study adds to our understanding of sociocultural factors that moderate these weight trends, particularly as it relates to the ethnic and generational composition of the social networks of Hispanic youth. Previous studies have shown that weight differences between immigrant generations become less evident when controlling for acculturation, but these studies have utilized broad measures of acculturation including neighborhood-level ethnic density, or proportion of foreign-born and Hispanic-identifying neighbors in an area (Gordon-Larsen et al., 2003). While such measures of acculturation provide insight about the cultural processes that potentially underlie generational weight trends, they assess neighborhood-level characteristics that may overlook more proximal and individual-specific processes. Our study filled this necessary gap by assessing the ethnic and generational composition of proximal social networks (i.e. friend groups) and their association with BMI across Hispanic generations.

The interaction of adolescents’ own generational status and the generational density of their social networks, specifically the proportion of second-generation Hispanics in their friend groups, suggests that social networks influence the BMI of Hispanic adolescents differently across generations, or alternatively, that the social networks of Hispanic adolescents are themselves influenced by generational BMI patterns. Prior research has described various generational trends in weight behaviors among Hispanics (Arandia et al., 2012; J. Liu et al., 2012a; J. Liu et al., 2009), and several qualitative studies of racial minority populations have suggested that proximal social networks may influence weight-behaviors through cultural and social pressures (Befort et al., 2008; Bertoni et al., 2011). Within this context, first-generation Hispanic adolescents with a peer social network that is predominantly composed of second-generation Hispanic adolescents may face unique cultural and social pressures to adopt acculturated behaviors that may subsequently impact BMI. Conversely, prior evidence suggests that individuals befriend others of similar weight (de la Haye et al., 2011; Powell et al., 2015). First-generation Hispanic adolescents with a relatively higher BMI may simply befriend other Hispanic adolescents with relatively higher BMIs, and those adolescents are more likely to be second or third-generation. Future studies should aim to assess these weights trends and relationships longitudinally to better understand their temporal relevance and causal associations.

Furthermore, the proportion of second-generation Hispanics in a social network, but not the proportion of first or third-generation friends, moderated the association between generational status and BMI. Second-generation Hispanics may be uniquely positioned to facilitate or impede the acculturative process in that they may share social and behavioral commonality with both immigrants and subsequent US-born generations. First-generation Hispanic adolescents may share more in common with second-generation than third-generation Hispanics, and thus are more likely to be influenced by the former but not the latter (Abraído-Lanza et al., 2016; Padilla and Perez, 2003). Of note, the significance of this interaction varied when adjusting for behavioral factors, in which sedentary activity and not MVPA were associated with BMI. These findings add to prior evidence suggesting that the association between MVPA and obesity is not consistently linear across sociodemographic contexts, and also alludes to the potential salience of sedentary activity as a mediator in the association between acculturation and BMI (Belcher et al., 2010; Byrd-Williams et al., 2007).

Finally, a social network with relatively more second-generation friends had opposing effects on first and second-generation Hispanic adolescents: it was associated with relatively higher BMI among first-generation Hispanics, but with lower BMI among those who were second-generation. These patterns may reflect the larger sociocultural context within which these networks reside, which are overall majority non-Hispanic and US-born, with relatively higher BMI. Second-generation Hispanic youth, who, consistent with prior literature, were more likely to have higher BMIs in our analyses, may influence first-generation Hispanic adolescents to embrace higher weights or to adopt unfavorable acculturated weight behaviors, while influencing second-generation Hispanic adolescents to retain lower weights or favorable, less acculturated behaviors (McCullough and Marks, 2014; Popkin and Udry, 1998). Alternatively, biased friend selection may have also contributed to these associations: first-generation friends who befriended second-generation friends may have been more likely to have higher weights, and second-generations friends who did not befriend similar generation friends may have themselves had relatively lower weights (Schaefer and Simpkins, 2014). Additionally, non-peer network factors including characteristics of family networks may be contributing to generational weight patterns (Mojica et al., 2019; Power et al., 2015).

### 4.1. Strengths and limitations

This study assessing the moderating role of the ethnic and generational composition of social networks on BMI patterns across Hispanic immigrant generations has several strengths, including a large and diverse sample of adolescents with thoroughly collected social network data. However, this study also has several limitations. The Add Health data analyzed was collected between 1994 and 1995 and there have been numerous political and social events since that time; notably, the association of time in the US, and generation, with weight have persisted, underscoring the continued need to understand the factors that influence these trends (Karen R Flórez et al., 2021). While Add Health is nationally representative, our analysis consisted specifically of the subsample of adolescents that were interviewed for the In Home survey and identified as Hispanic, precluding the use of survey weights. Our analysis also consisted of cross-sectional data, and while we found significant interactions between social network characteristics and generational status as it relates to BMI, causal inferences cannot be made. Our analysis does not account for differences in social networks over time or time since migration. To retain concordance in time-collection between our analyzed variables, our analysis calculated BMI based on self-reported rather than objectively-measured weight and height, the latter of which was not collected at the time that the social network variables were collected. There are numerous measures of acculturation used in the literature, and a considerable amount of efforts continue to explore the optimal measurement of this complex multi-dimensional concept. Though several studies of acculturation have specifically focused on generational status (Bates et al., 2008; Karen R. Flórez et al., 2019; Gordon-Larsen et al., 2003; Guarini et al., 2011; Harris et al., 2009; Peña et al., 2008; Popkin and Udry, 1998; Singh et al., 2009) generational status is not a direct measure of acculturation and Add Health questionnaires did not allow for a more nuanced or multi-faceted assessment of acculturation. Additionally, there was considerable missingness in certain sociodemographic variables, including household income, limiting our ability to include these variables in our analysis. Finally, the behavioral variables (physical and sedentary activity) used self-reported data. Future studies should aim to objectively measure anthropomorphic data and behavioral data through accelerometry, while also assessing social network variables longitudinally.

## 5. Conclusion

The generational status of peers in Hispanic adolescent’s social networks, particularly the number of second-generation Hispanics, moderates the positive association between immigrant generation and BMI. Moreover, this moderation effect is different across immigrant generations so that the proportion of second-generation adolescents within a social network is associated with higher BMI in first-generation Hispanic adolescents, but with lower BMI among those who are second-generation. Our findings suggest that social networks may potentially influence weight differently across immigrant generations. Future research should aim to longitudinally assess the impact of generational status on weight and to further elucidate proximal social factors affecting the weight and weight behaviors of vulnerable immigrant populations.

## Supplementary Material

Supplementary Table 1. Multivariable Analyses of Body Mass Index among Hispanic Adolescents of Add Health, using Multiple Imputation

## Figures and Tables

**Table 1:** Characteristics of Hispanic adolescents in wave 1 of add health.

	Total Hispanic Sample		First-Generation		Second Generation		Third Generation	
	n/mean	%/SD	n/mean	%/SD	n/mean	%/SD	n/mean	%/SD
Sample Size, n	1950	100%	543	27.9%	916	47.0%	491	25.2%
**Demographics**								
Male, n	986	50.6%	274	50.5%	458	50.0%	254	51.7%
Age, mean	16	1.65	16.46	1.53	15.95	1.62	15.6	1.71
Hispanic Origin, n								
Mexican/Chicano	823	42.4%	134	24.7%	435	47.5%	254	51.7%
Cuban	420	21.6%	202	37.2%	202	22.1%	16	3.3%
Puerto Rican	382	19.7%	38	7.0%	175	19.1%	169	34.4%
Central/South American	201	10.4%	127	23.4%	65	7.1%	9	1.8%
Other Hispanic	115	5.9%	42	7.7%	36	3.9%	37	7.5%
Parent received public assistance, n	267	13.7%	102	18.8%	126	13.8%	39	7.9%
**Social Network Characteristics**								
Isolate, n^a^	103	5.30%	40	7.4%	45	4.9%	18	3.7%
Total size, mean	5.82	3.93	5.22	3.73	5.83	3.96	6.47	4.01
Total Hispanic size, mean	3.56	3.24	4.03	3.32	3.93	3.29	2.35	2.70
Proportion Total Network Hispanic, mean^a^	0.65	0.37	0.82	0.31	0.71	0.33	0.38	0.34
Proportion First-generation Hispanic, mean^a^	0.23	0.31	0.47	0.35	0.19	0.26	0.05	0.15
Proportion Second-generation Hispanic, mean^a^	0.27	0.29	0.24	0.27	0.36	0.31	0.14	0.21
Proportion Third-generation Hispanic, mean^a^	0.07	0.14	0.03	0.1	0.07	0.14	0.1	0.17
**Outcomes**								
Body mass index (kg/m^2^), mean	23.2	4.54	22.75	3.98	23.47	4.79	23.20	4.60
Total bouts of MVPA/week (0–18), mean^b^	6.45	4.18	6.12	4.21	6.41	4.18	6.91	4.14
Total sedentary hours/week (0–282), mean^c^	22.34	20.32	21.40	21.48	23.05	20.06	22.05	19.47

MVPA: Moderate to Vigorous Physical Activity (5–8 metabolic equivalents).

a Isolates (adolescents with total network size = 0) were not included in calculations of network proportion means, n = 1847.

b Total bouts of MVPA were determined by summation of responses to the questions “During the past week how many times did you …” 1) riding bike, roller-skating or rollerblading, 2) participation in active sports such as soccer, baseball, or basketball, and 3) active exercises such as walking, running, dancing, or jumping rope.

c Sedentary activity was measured as the self-reported hours per week that adolescents performed 3 activities: 1) viewing television, 2) viewing video cassettes and 3) computer/video game use.

**Table 2:** Multivariable analyses of body mass index among Hispanic adolescents of add health.

	Model 1		Model 2		Model 3		Model 4		Model 5		Model 6		Model 7		Model 8	
	β	SE	β	SE	β	SE	β	SE	β	SE	β	SE	β	SE	β	SE
**Generational Status**																
First-generation	REF	REF	REF	REF	REF	REF	REF	REF	REF	REF	REF	REF	REF	REF	REF	REF
Second-generation	***0.94*** ***	***0.24***	***0.94*** ***	***0.24***	***1.66*** **	***0.58***	***0.78*** **	***0.27***	***0.72*** *	***0.35***	***1.36*** ***	***0.36***	***0.76*** **	***0.29***	***1.26*** ***	***0.36***
Third-generation	***0.88*** **	***0.28***	***0.84*** **	***0.31***	0.97	0.52	***0.74*** *	***0.33***	0.62	0.37	***1.17*** **	***0.40***	0.72	0.37	***1.13*** **	***0.40***
**Sociodemographic Variables**																
Age	***0.44*** ***	***0.07***	***0.43*** ***	***0.07***	***0.44*** ***	***0.07***	***0.43*** ***	***0.07***	***0.44*** ***	***0.07***	***0.44*** ***	***0.07***	***0.44*** ***	***0.07***	***0.48*** ***	***0.07***
Sex, male	***0.65*** **	***0.21***	***0.63*** **	***0.21***	***0.62*** **	***0.21***	***0.64*** **	***0.21***	***0.64*** **	***0.21***	***0.64*** **	***0.21***	***0.64*** **	***0.21***	***0.55*** **	***0.21***
Parent received public assistance	0.49	0.34	0.45	0.34	0.46	0.34	0.46	0.34	0.46	0.34	0.45	0.34	0.46	0.34	0.33	0.33
**Social Network Characteristics**																
Total Network Size			−0.05*	***0.03***	−0.05*	***0.03***	−0.06*	***0.03***	−0.06*	***0.03***	−0.06*	***0.03***	−0.06*	***0.03***	−0.06*	***0.03***
Prop. of Network Hispanic			−0.19	0.33												
First-gen x Prop. of Network Hispanic					0.21	0.49										
Second-gen x Prop. of Network Hispanic					−0.94	0.70										
Third-gen x Prop. of Network Hispanic					0.15	0.79										
Proportion of Network First-gen							−0.70	0.42			−0.55	0.43	−0.71	0.43	−0.65	0.42
First-gen x Prop. Network First-gen									−0.85	0.52						
Second-gen x Prop. Network First-gen									0.15	0.83						
Third-gen x Prop. Network First-gen									0.90	1.53						
Proportion of Network Second-gen							0.27	0.41	0.24	0.41			0.27	0.41		
First-gen x Prop. Network Second-gen											***1.64*** *	***0.73***			***1.50*** *	***0.73***
Second-gen x Prop. Network Second-gen											−1.92*	***0.88***			−1.73*	***0.87***
Third-gen x Prop. Network Second-gen											−1.59	1.22			−1.49	1.20
Proportion of Network Third-gen							−1.06	0.68	−1.09	0.68	−1.13	0.68			−1.17	0.69
First-gen x Prop. Network Third-gen													−1.60	1.60		
Second-gen x Prop. Network Third-gen													0.66	1.85		
Third-gen x Prop. Network Third-gen													0.50	1.91		
**Behavioral Factors**																
Total bout of MVPA/week, mean															0.00	0.03
Total sedentary hours/week, mean															***0.02*** **	***0.01***

* **p < 0.05**,

** **p < 0.01**,

*** **p < 0.001**.

Significant findings are bolded and italicized for clarity. MVPA: Moderate to vigorous physical activity. “Gen” is generation. “Prop. of Network Hispanic” is the proportion of the adolescent’s friends that identify as Hispanic. “Prop. Network X-gen” are the proportion of the adolescent’s friends that identify of a X generational status. N = 1847 for all models.

## Data Availability

The data that has been used is confidential.

## Appendix A. Supplementary data

Supplementary data to this article can be found online at https://doi.org/10.1016/j.socscimed.2023.115831.
