# Supplementary material for: Associations of peer generational status on adolescent weight across Hispanic immigrant generations: A social network analysis
Source: Soc Sci Med. Author manuscript; Available in PMC 2025 Dec 19. (PMC12715723; doi:10.1016/j.socscimed.2023.115831)
Supplement: Supplementary Table 1. Multivariable Analyses of Body Mass Index among Hispanic Adolescents of Add Health, using Multiple Imputation [file NIHMS2123638-supplement-Supplementary_Table_1__Multivariable_Analyses_of_Body_Mass_Index_among_Hispanic_Adolescents_of_Add_Health__using_Multiple_Imputation.pdf]

Supplementary Table 1. Multivariable Analyses of Body Mass Index among Hispanic Adolescents of Add Health, using Multiple Imputation

	Model 1		Model 2		Model 3		Model 4		Model 5		Model 6		Model 7		Model 8	
	β	SE	β	SE	β	SE	β	SE	β	SE	β	SE	β	SE	β	SE
**Generational Status**																
First-generation	REF	REF	REF	REF	REF	REF	REF	REF	REF	REF	REF	REF	REF	REF	REF	REF
Second-generation	0.97***	0.23	0.96***	0.23	1.71**	0.57	0.82**	0.27	0.77*	0.35	1.37***	0.36	0.78**	0.29	1.27***	0.35
Third-generation	0.83**	0.27	0.82**	0.30	0.97	0.51	0.73*	0.32	0.63	0.37	1.16**	0.40	0.70	0.37	1.11**	0.39
**Sociodemographic Variables**																
Age	0.44***	0.06	0.43***	0.07	0.43***	0.07	0.42***	0.07	0.43***	0.07	0.43***	0.07	0.42***	0.07	0.47***	0.07
Sex, male	0.62**	0.20	0.59**	0.20	0.58**	0.20	0.60**	0.20	0.60**	0.20	0.61**	0.20	0.60**	0.20	0.50*	0.21
Parent received public assistance	0.53	0.33	0.46	0.33	0.46	0.33	0.46	0.33	0.46	0.33	0.45	0.33	0.46	0.33	0.33	0.32
**Social Network Characteristics**																
Total Network Size			-0.06*	0.03	-0.06*	0.03	-0.06*	0.03	-0.06*	0.03	-0.06*	0.03	-0.06*	0.03	-0.06*	0.03
Prop of Total Network Hispanic			-0.21	0.33	0.22	0.49										
First-gen x Prop Network Hispanic																
Second-gen x Prop Network Hispanic					-0.98	0.69										
Third-gen x Prop Network Hispanic					0.12	0.78										
Prop of Total Network First-gen Hispanic							-0.68	0.42			-0.52	0.42	-0.69	0.42	-0.62	0.42
First-gen x Prop Network First-gen									-0.79	0.52						
Second-gen x Prop Network First-gen									0.09	0.82						
Third-gen x Prop Network First-gen									0.87	1.53						
Prop of Total Network Second-gen Hispanic							0.23	0.40	0.21	0.40			0.23	0.41		
First-gen x Prop Network Second-gen											1.58*	0.72			1.40	0.72
Second-gen x Prop Network Second-gen											-1.86*	0.87			-1.63	0.86
Third-gen x Prop Network Second-gen											-1.61	1.20			-1.49	1.19
Prop of Total Network Third-gen Hispanic							-1.13	0.67	-1.14	0.67	-1.17	0.67			-1.23	0.67
First-gen x Prop Network Third-gen													-1.74	1.59		
Second-gen x Prop Network Third-gen													0.70	1.83		
Third-gen x Prop Network Third-gen													0.70	1.90		
**Behavioral Factors**																
Total bout of MVPA/week, mean															0.00	0.02
Total sedentary hours/week, mean															0.02**	0.01

*p<0.05, ** p<0.01, ***p<0.001. Significant findings are bolded and italicized for clarity. "Gen" is generation. "Prop. of Network Hispanic" is the proportion of the adolescent's friends that identify as Hispanic. "Prop. Network X-gen" are the proportion of the adolescent's friends that identify of a X generational status. N=1876 for all models.
